# Meta-Analysis of Dexmedetomidine on Emergence Agitation and Recovery Profiles in Children after Sevoflurane Anesthesia: Different Administration and Different Dosage

**DOI:** 10.1371/journal.pone.0123728

**Published:** 2015-04-13

**Authors:** Min Zhu, Haiyun Wang, Ai Zhu, Kaijun Niu, Guolin Wang

**Affiliations:** 1 Department of Anesthesiology, Tianjin Research Institute of Anesthesiology, Tianjin Medical University General Hospital, Tianjin 300052, China; 2 Department of Epidemiology, School of Public Health, Tianjin Medical University, Tianjin 300052, China; Central South University, CHINA

## Abstract

The objective of this article is to evaluate the effect of dexmedetomidine on emergence agitation (EA) and recovery profiles in children after sevoflurane anesthesia and its pharmacological mechanisms. Standard bibliographic databases, including MEDLINE, EMBASE, PsycINFP, Springer and ISI Web of Knowledge, were artificially searched to identify all randomized controlled trials (RCTs) comparing the impact of dexmedetomidine with placebo, fentanyl and midazolam on EA and recovery profiles after sevoflurane anesthesia in post-anesthesia care unit (PACU). Two authors assessed the quality of each study independently in accordance with strict inclusion criteria and extracted data. RevMan 5.0 software was applied for performing statistic analysis. The outcomes analyzed included: 1) incidence of EA, 2) emergence time, 3) time to extubation, 4) incidence of post-operation nausea and vomiting, 5) number of patients requiring an analgesic, and 6) time to discharge from PACU. A total of 1364 patients (696 in the dexmedetomidine group and 668 in the placebo, fentanyl and midazolam group) from 20 prospective RCTs were included in the meta-analysis. Compared with placebo, dexmedetomidine decreased the incidence of EA (risk ratio [RR] 0.37; 95% CI 0.30 to 0.46), incidence of nausea and vomiting (RR 0.57; 95% CI 0.38 to 0.85) and number of patients requiring an analgesic (RR 0.43; 95% CI 0.31 to 0.59). However, dexmedetomidine had a significantly delayed effect on the emergence time (weighted mean differences [WMD] 1.16; 95% CI 0.72 to 1.60), time to extubation (WMD 0.61; 95% CI 0.27 to 0.95), and time to discharge from recovery room (WMD 2.67; 95% CI 0.95 to 4.39). Compared with fentanyl (RR 1.39; 95% CI 0.78 to 2.48) and midazolam (RR 1.12; 95% CI 0.54 to 2.35), dexmedetomidine has no significantly difference on the incidence of EA. However, the analgesia effect of dexmedetomidine on postoperation pain has no significantly statistical differences compared with fentanyl (RR 1.12; 95% CI 0.66 to 1.91), which implied that its analgesia effect might play an important role in decreasing the incident of EA. No evidence of publication bias was observed.

## Introduction

Children undergoing elective surgeries such as strabismus, tonsillectomy and outpatient surgeries, especially under sevoflurane anesthesia, often experience emergency agitation (EA) and other uncomfortable symptoms [1,2]. EA is a postoperative negative behavior that may include symptoms such as combative movements, excitability, thrashing, disorientation and inconsolable crying [3]. In addition, it is a troublesome phenomenon that can result in injury to the children themselves or damage to the surgical site, leading to dissatisfaction and anxiety for the parents and requiring extra nursing care, which further increases associated healthcare costs [2]. Some studies found that EA was closely associated with various etiologies such as anxiety, pain, physiologic compromise and anesthetics [4]. Moreover, nausea and vomiting, severe pain, eye opening time and extubation time are also the main factors for discharge from the post-anesthesia care unit (PACU) or hospital, affecting the children’s recovery [5]. Therefore, anesthesiologists aim to reduce the incidence of EA and improve the quality of children’s postoperative condition with the help of various drugs and techniques.

Although different drugs including opioids, benzodiazepines and clonidine, one kind of α_2_-adrenoreceptor agonists, have been tried in clinic either as a prophylactical measure or treatment, the results have been variable[6–10]. Among which, dexmedetomidine, a highly specific α_2_-adrenoreptor agonist (receptor selectivity, α_2_/α_1_ = 1620/1), has sedative and analgesic properties without significant respiratory depression at the clinically approved dosage [11,12]. As the result of it, dexmedetomidine has been administered perioperatively to reduce postoperative negative behaviors such as EA and aggression. However it is notable that several study results remain ambiguous about the effectiveness and safety of dexmedetonidine with regard to children’s postoperative condition [2, 13–31].

Therefore, the aim of this study was to undertake a systematic review of the literature to perform a meta-analysis to determine the influence of dexmedetonidine administration on EA and post-operative recovery profiles in children after sevoflurane anesthesia.

## Materials and Methods

### Search strategy

The methods of this meta-analysis were performed in accordance with the Cochrane Collaboration criterion. MEDLINE, EMBASE, PsycINFP, Springer and ISI Web of Knowledge were searched for relevant electronic studies of randomized controlled trials (RCTs) published before October 31^th^, 2014. Hand searching techniques also were used to identify appropriate studies. Moreover, we used the following search terms, dexmedetonidine, children, and EA, and limited the search strategy to English language reports in humans. Articles that met the following criteria were included: 1) randomized and double-blind study design; 2) the intervention was treatment with dexmedetonidine given systemically in any dose during the perioperative period; 3) children undergoing sevoflurane anesthesia experiencing EA and 4) participants had no preoperative cognitive dysfunction, had not undergone neurosurgery and had not received pharmacological intervention which disturbs cognitive function, such as epilepsy drugs.

### Data extraction and analysis

All data were extracted by two reviewers (H. Wang and G. Wang) independently reviewing every selection for accuracy and consistency. The following outcome measures were extracted from the retrieved reports in the form of mean data plus standard deviation or dichotomous data: 1) incidence of EA, 2) the emergence time, 3) time to extubation, 4) incidence of post-operation nausea and vomiting, 5) number of patients requiring an analgesic and 6) time to discharge from recovery room. Moreover, the subgroup analysis was performed: 1) different administration ways, 2) different drug dosage, and 3) different surgery procedures. Meanwhile the effect of dexmedetomidine on the incidence of EA was compared following anesthesia induced with fentanyl and midazolam. Furthermore, the analgesic effect of dexmedetomidine was also compared with that of fentanyl. If the necessary data did not allow inclusion in the meta-analysis, the authors of those papers were contacted directly and asked to release the relevant data. Quality of the included studies was assessed by using the modified Jadad score [32] including four items pertaining to description of randomization, allocation concealment, appropriate blinding, and dropouts or withdrawals. The final selected studies were reviewed by an expert (K. Niu) on statistics to ensure the thoroughness and completeness.

### Statistical analysis

Statistical analysis was performed using the Review Manager 5 software. Dichotomous data were analyzed by using the risk ratio (RR) computed using the Mantel Haenszel method (fixed or random models). Continuous outcomes measured on the same scale were expressed as a mean value and standard deviation and were analyzed by using weighted mean differences (WMD). I-square (I^2^) test was performed to assess the impact of study heterogeneity on the results of the meta-analysis. According to the Cochrane review guidelines, if severe heterogeneity was present at I^2^ >50%, the random effect models were chosen, otherwise the fixed effect models were used. Moreover, sensitivity analysis was conducted by deleting each study individually to evaluate the quality and consistency of the results. The funnel plot was used to detect potential publication bias.

## Results

### Outcomes

**Fig 1:**
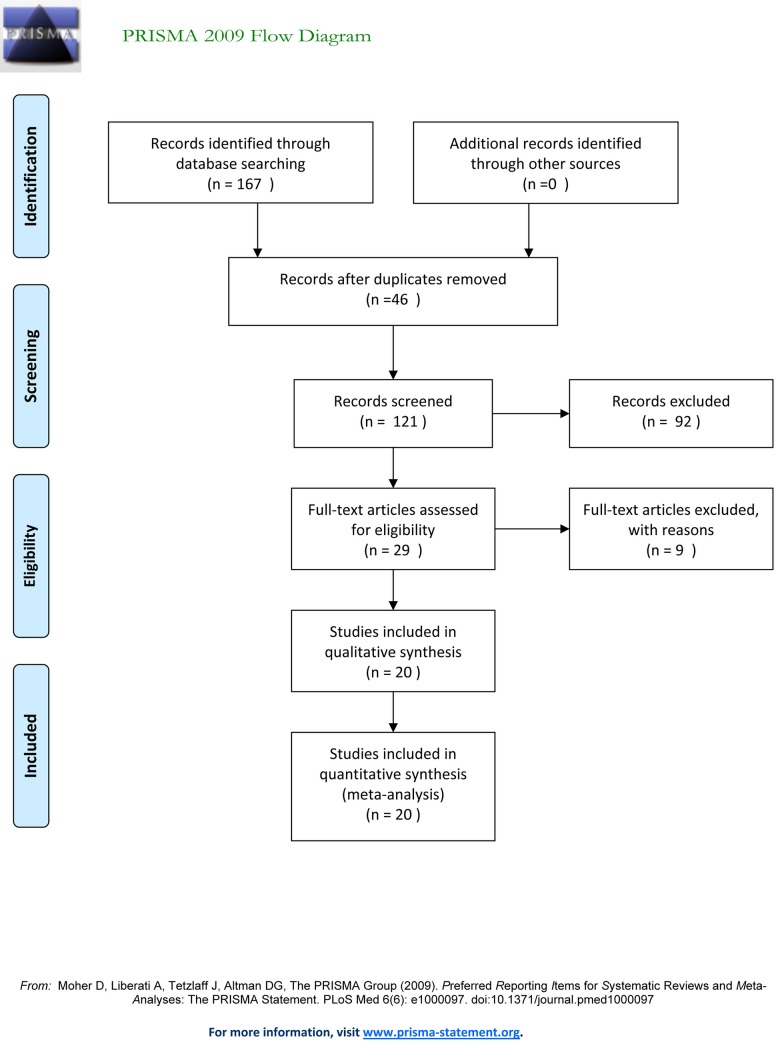
Studies eligible for inclusion in meta-analysis.

The vast majority of the literature search results were excluded due to an inappropriate study design, population, intervention or outcome measure. Subsequently, 20 RCTs were included in this meta-analysis (Fig 1). The characteristic of included studies including intervention, premedication, surgery procedures, anesthesia induction and maintenance of anesthesia were collected (Table 1). Moreover our team workers evaluated the quality of included studies by using Modified Jadad Scores (Table 2). To each analysis, the heterogeneity, the model to pool (random effect or fixed effect), the pooled result, and the P value also were showed (Table 3).

**Table 1:** Characteristics of included studies.

Author	Year	Age(y)	Intervention	Dexmedetomidine	Surgery	ASA	Pre-medication	Anethesia induce	Anethesia
			(No.of patients)	Dose(Time)					maintain
Sato M[2]	2010	1–9y	Dexmedetomidine(39)	0.3μg·kg^-1^ iv over	same-day surgery or	ⅠⅡ	NO	8% sevoflurane in	2–5% sevoflurane
				10min after induction of	over-night stay				in 2 L·min-1 O_2_
			/Placebo(42)	anesthesia	surgery			6 L·min^-1^ O_2_	and 4 L·min^-1^ air
		
Chen JY[13]	2013	2–7y	Dexmedetomidine(28)	1.0μg·kg^-1^ iv plus	elective strabismus	ⅠⅡ	NO	O_2_ (F_1_O_2_ = 1.0,	oxygen (F_1_O_2_ = 1.0,
								5L·min^-1^) and 8%	5L·min^-1^) and 8%
			/Placebo(28)	1.0μg·kg^-1^·h^-1^	surgery			sevoflurane	sevoflurane
	
Isik B[14]	2006	1.5–10y	Dexmedetomidine(21)	1.0μg·kg^-1^ iv after	MRI	ⅠⅡ	NO	8% sevoflurane in	1.5–2.0%
								2.5 L·min^-1^ N_2_O and	sevoflurane in
			/Placebo(21)	induction of anesthesia				2.5 L·min^-1^ O_2_	50/50%
									N_2_O and O_2_
Meng QT[15]	2012	5–14y	Dexmedetomidine(80)	0.5μg·kg^-1^ iv plus	tonsillectomy	ⅠⅡ	40μg·kg^-1^	pro 1.5–2.0 mg·kg^-1^	1.5–2.5% sev in
				0.2μg·kg^-1^·h^-1^				sufentani 0.4 μg·kg^-1^	2.0L·min^-1^ O_2_0.5-
			/Placebo(40)	or 1.0μg·kg^-1^ iv plus	operation		iv imidazole	and cisatracurium	1.0μg·kg^-1^·min^-1^
				0.4μg·kg^-1^·h^-1^				0.15 mg·kg^-1^	remifentanil
Shukry M[16]	2005	1–10y	Dexmedetomidine(23)	0.2μg·kg^-1^·h^-1^ iv started	elective outpatient	ⅠⅡ	NO	8% sevoflurane in O_2_	sevoflurane
								and spontaneous	concentration to
			/Placebo(23)	after securing airway	surgical procedures			ventilation	achieve 40–60 BIS
									and fentanyl or not
Ibacache ME[17]	2004	1–10y	Dexmedetomidine(60)	0.15μg·kg^-1^ after	superficial lower	Ⅰ	NO	50% N_2_O and 8%	1% sevoflurane
				induction of anesthesia	abdominal and genital			sevoflurane in O_2_	end-tidal in 50%
			/Placebo(30)	or 0.30μg·kg^-1^	surgery			and spontaneous	N_2_O
								ventilation	
Guler G[18]	2005	3–7y	Dexmedetomidine(30)	0.5μg·kg^-1^ iv before the	adenotonsillectomy	Ⅰ	acetaminophen	50% N_2_O and 8%	1.5–2.0% in 60%
							15mg·kg^-1^ oral	sevoflurane in O_2_	N_2_O and 40% O_2_
			/Placebo(30)	end of surgery			before induce	and spontaneous	with controlled
								ventilation	ventilation
Xu LL[19]	2012	3–7y	Dexmedetomidine(30)	0.5μg·kg^-1^ iv over 10	vitreoretinal surgery	ⅠⅡ	NO	8% sevoflurane in O_2_	remifentani 0.2
				min after induction of				and spontaneous	μg·kg^-1^·min-1 and
			/Placebo(30)	anethesia				ventilation	1–2% end-tidal
									sevoflurane in O_2_
Pestieau SR[20]	2011	0.5–6y	Dexmedetomidine(51)	1.0μg·kg^-1^ or 2.0μg·kg^-1^	BMT	ⅠⅡ	NO	N_2_O/O_2_ (2:1) gas	2–3% sevoflurane
				by nasal mucosa after				mixture and	inspired
			/Placebo(27)	induction of anethesia				sevoflurane	concentration and
									N_2_O:O_2_ mixture
Kim NY[21]	2014	1–6y	Dexmedetomidine(20)	1.0μg·kg^-1^ iv plus	ambulatory surgery	Ⅰ	NO	6–7% sevoflurane in	adjust end-tidal
									sevoflurane
			/Placebo(20)	0.1μg·kg-1·h^-1^				4 L/min O_2_	concentration to
									achieve 45–50 BIS
GF EI-Rahmawy[22]	2013	2–6y	Dexmedetomidine(14)	2.0μg·kg^-1^ by fascia	farcture femur surgery	ⅠⅡ	0.5mg·kg^-1^	1%-5% increasing	sevoflurane 1–1.5
				iliaca copartment block			imidazole 1h prior	sevoflurane	MAC in 40%
			/Placebo(14)	after induction of			to induction	concentration in	air/O_2_ mixture
				anesthesia				100% O_2_	
OM Asaad[23]	2011	5–10y	Dexmedetomidine(30)	0.15μg·kg^-1^ iv after	elective surgical	Ⅰ	NO	50% N_2_O and	1% end-tidal
								sevoflurane up to 8%	sevoflurane in
			/Placebo(30)	induction of anesthesia	procedures			in O_2_ with total gas	50% N_2_O
								above 5L/min	
Saadawy I[24]	2009	1–6y	Dexmedetomidine(30)	1.0μg·kg^-1^ by caudal	unilateral inguinal	Ⅰ	NO	propofol 3–4mg·kg^-1^	0.5–2.0
				block after induction of	hernia				sevoflurane and
			/Placebo(30)	anesthesia	repair /orchidopexy				70% N_2_O in O_2_
		
He L[25]	2013	3–7y	Dexmedetomidine(61)	0.5μg·kg^-1^ or 1.0μg·kg^-1^	elective minor	ⅠⅡ	NO	8% sevoflurane in O_2_	2–3% sevoflurane
								via a semiclosed	in 1 L/min O_2_ and
			/Placebo(26)	iv after LMA insertion	surface surgery			anaeshetic	1 L/min air
	
Erdil F[26]	2009	2–7y	Dexmedetomidine(30)	0.5μg·kg^-1^ iv after	adenotonsillectomy	Ⅰ	paracetamol 40	50% N_2_O and 8%	1.5–2.5%
					with or without		mg·kg^-1^ reactally		sevoflurane in
			/Placebo(30)	tracheal intubation	bilateral myringotomy		60min before	sevoflurane in O_2_	70% N_2_O/O_2_
							induction		
Gupta N[27]	2013	8–12y	Dexmedetomidine(18)	1.0μg·kg^-1^ iv over ten	corrective surgery	ⅠⅡ	intramuscular	8% sevoflurane;	60% N_2_O and 3
				minutes			glycopyrrolate	1mg·kg^-1^ rocuronium	L/min sevoflurane
			/Placebo(18)	plus 0.5μg·kg^-1^·h^-1^			0.2mg 1h before	and 2ug·kg^-1^	to maintain EtCO_2_
							induction	fentanyl	of 35–40 mmHg
Ozcengiz D[28]	2011	3–9y	Dexmedetomidine(25)	2.5μg·kg^-1^ by oral	esophageal dilatation	ⅠⅡ	paracetamol	8% sevoflurane and	2–4% sevoflurane
				before induction of			2–2.5mg·kg^-1^		
			/Placebo(25)	anesthesia			before induction	50% N_2_O in O_2_	and 50% N_2_O
	
Ali MA[29]	2013	2–6y	Dexmedetomidine(40)	0.3μg·kg^-1^ iv before the	adenotonsillectomy	ⅠⅡ	0.5 mg·kg^-1^	8% sevoflurane in	60% N_2_O and
				end of surgery over			midazole oral		2–3% sevoflurane
			/Placebo(40)	five minutes			30 min before	70% N_2_O in O_2_	to EtCO_2_ of
							induction		35±4mmHg
Mountain BW[30]	2011	1–6y	Dexmedetomidine(22)	4μg·kg^-1^ by oral before	dental restoration and	ⅠⅡ	NO	sevoflurane oxygen	isoflurane for
					possible tooth				
			/Midazolam(19)	induction of anesthesia	extraction			and nitrous oxide	mainenance

Akin A[31]	2013	2–9y	Dexmedetomidine(45)	1μg·kg^-1^ intranasal	adenotonsillectomy	Ⅰ	NO	sevoflurane oxygen	sevoflurane to
				approximately 45–60					maintain EtCO_2_ of
			/Placebo(45)	min before the induction				and nitrous oxide	35–40mmHg
				of anesthesia					

MRI: magnetic resonance imaging; IV: intravenous; BMT: bilateral myringotomy; LMA: the laryngeal mask airway; ASA: American Society of Anesthesiologists physical status; BIS: Bispectral Index Score; MAC: minimum alveolar concentration; EtCO2: end-tidal carbon dioxide

**Table 2:** The modified Jadad scores.

Author	Randomization	Allocation concealment	Binding	Withdrawl or dropouts	Total scores
Sato M[2]	randomization list (2)	just described (1)	double-blind (2)	Yes(1)	5
Chen JY[13]	computer-generated (2)	central randomization (2)	double-blind (2)	No(0)	6
Isik B[14]	randomization list (2)	central randomization (2)	double-blind (2)	No(0)	6
Meng QT[15]	computer-generated (2)	central randomization (2)	double-blind (2)	No(0)	6
Shukry M[16]	computer-generated (2)	just described (1)	double-blind (2)	No(0)	5
Ibacache ME[17]	computer-generated (2)	just described (1)	double-blind (2)	No(0)	5
Guler G[18]	computer-generated (2)	just described (1)	double-blind (2)	No(0)	5
Xu LL[19]	computer-generated (2)	central randomization (2)	double-blind (2)	No(0)	6
Pestieau SR[20]	computer-generated (2)	central randomization (2)	double-blind (2)	No(0)	6
Kim NY[21]	computer-generated (2)	just described (1)	double-blind (2)	No(0)	5
GF EI-Rahmawy[22]	computer-generated (2)	central randomization (2)	double-blind (2)	No(0)	6
OM Asaad[23]	concealed envelope method (2)	just described (1)	double-blind (2)	No(0)	5
Saadawy I[24]	computer-generated (2)	central randomization (2)	double-blind (2)	No(0)	6
He L[25]	computer-generated (2)	central randomization (2)	double-blind (2)	Yes(1)	7
Erdil F[26]	computer-generated (2)	central randomization (2)	double-blind (2)	No(0)	6
Gupta N[27]	computer-generated (2)	just described (1)	double-blind (2)	No(0)	5
Ozcengiz D[28]	table random method (2)	just described (1)	double-blind (2)	No(0)	5
Ali MA[29]	computer-generated (2)	central randomization (2)	double-blind (2)	No(0)	6
Mountain BW[30]	computer-generated (2)	central randomization (2)	double-blind (2)	No(0)	6
Akin A[31]	computer-generated (2)	central randomization (2)	double-blind (2)	No(0)	6

**Table 3:** Statistics information of each analysis.

Analysis	Heterogeneity	Model of pool	Pooled result	P value
Dex vs Placebo				
1. Incidence of EA	I^2^ = 2%	Fixed effect	95% CI 0.37(0.30, 0.46)	P<0.00001
Subgroup				
1.1 different administration				
intravenous	I^2^ = 0%	Fixed effect	95% CI 0.36(0.28, 0.45)	P<0.00001
intrathecal	I^2^ = 0%	Fixed effect	95% CI 0.19(0.06, 0.60)	P = 0.005
mucocutaneous	I^2^ = 6%	Fixed effect	95% CI 0.55(0.33, 0.93)	P = 0.02
1.2 different surgery procedure				
outpatient surgery	I^2^ = 10%	Fixed effect	95% CI 0.32(0.22, 0.46)	P<0.00001
E.N.T surgery	I^2^ = 0%	Fixed effect	95% CI 0.47(0.33, 0.66)	P<0.00001
selective surgery	I^2^ = 15%	Fixed effect	95% CI 0.32(0.21, 0.49)	P<0.00001
1.3 bolus or continuous dosage				
bolus dosage	I^2^ = 8%	Fixed effect	95% CI 0.38(0.29, 0.48)	P<0.00001
continuous dosage	I^2^ = 0%	Fixed effect	95% CI 0.35(0.22, 0.54)	P<0.00001
2. Time to eye opening	I^2^ = 0%	Fixed effect	95% CI 1.27(0.73, 1.82)	P<0.00001
3. Time to extubation	I^2^ = 26%	Fixed effect	95% CI 0.61(0.27, 0.95)	P = 0.0004
4. Incidence of post-operation vomiting	I^2^ = 0%	Fixed effect	95% CI 0.57(0.38, 0.85)	P<0.006
5. Number of patients requiring analgesic	I^2^ = 0%	Fixed effect	95% CI 0.43(0.31, 0.59)	P<0.00001
6. Time to discharge from PACU	I^2^ = 0%	Fixed effect	95% CI 2.67(0.95, 4.39)	P = 0.002
Dex vs Fentanyl				
Incidence of EA	I^2^ = 0%	Fixed effect	95% CI 1.39(0.78, 2.48)	P = 0.26
Dex vs Midazolam				
Incidence of EA	I^2^ = 3%	Fixed effect	95% CI 1.12(0.54, 2.35)	P = 0.76

Dex:dexmedetomidine; PACU: post-anesthesia care unit

### Risk of bias

**Fig 2:**
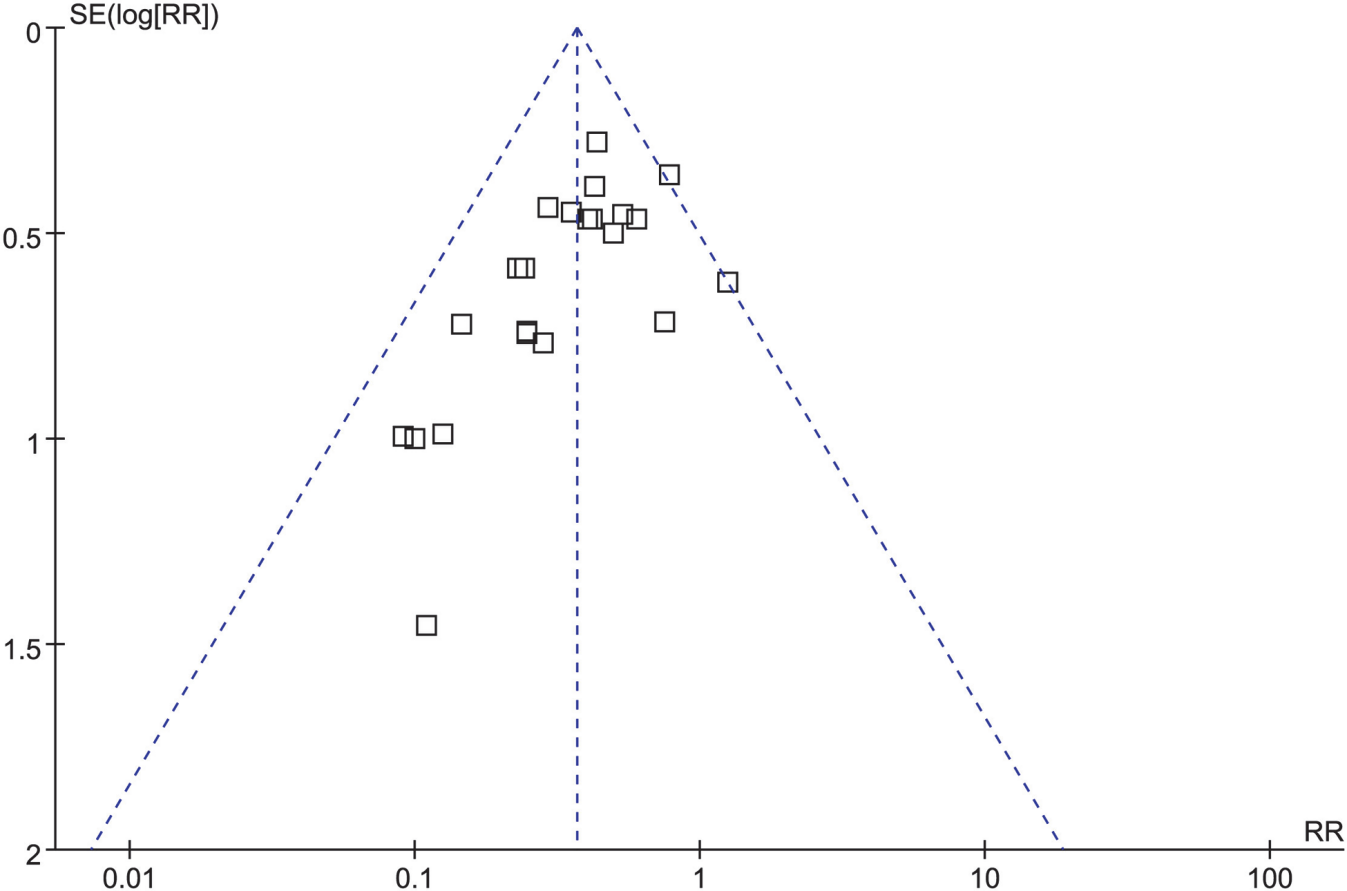
Funnel plot of the studies included in the meta-analysis. The vertical line represents the meta-analysis summary estimate, and the scatter represents single studies. In the absence of publication bias, studies will be distributed symmetrically right and left of vertical line. Log risk ratio (RR), natural logarithm of the RR; SE (log RR), standard error of the log RR.

The funnel plot was applied for assessing publication bias of studies included in the incidence of EA in this meta-analysis. No evident publication bias was obtained through the visual distribution of funnel plot (Fig 2).

### Sensitivity analysis

We evaluated the effect of each study on the pooled results by excluding single study sequentially. The result shown that the stability of results had no significant changes (data not shown), which validated the rationality and reliability of our analysis.

### Incidence of EA

**Fig 3:**
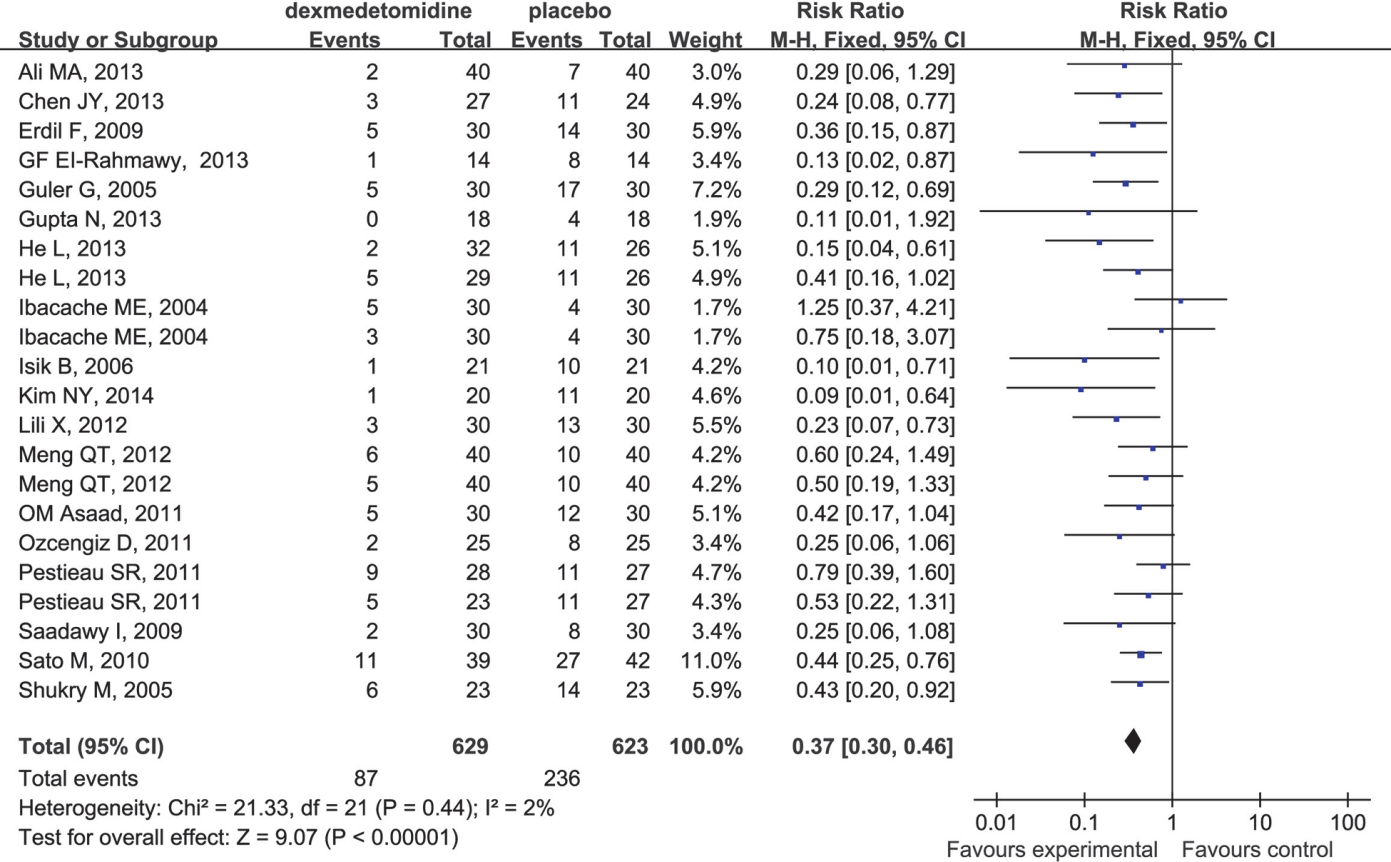
Forest plot for the incidence of EA. The plot displays the study, sample size, weighted risk ratio (RR), confidence interval (CI), and P value. Meta-analysis indicates dexmedetomidine significantly decreased the incidence of EA compared with placebo. The square shown for each study (first author and year of publication) is the RR for individual trials, and the corresponding horizontal line is the 95% CI. The diamond is the pooled RR with the CI. The different sizes of squares indicate the weight the individual trials had in the analysis, taking into account sample size.

Data extracted from relevant articles indicated that dexmedetomidine compared to placebo significantly decreased the incidence of EA in children undergoing sevoflurane anesthesia (RR 0.37; 95%CI 0.30 to 0.46; Fig 3).

**Fig 4:**
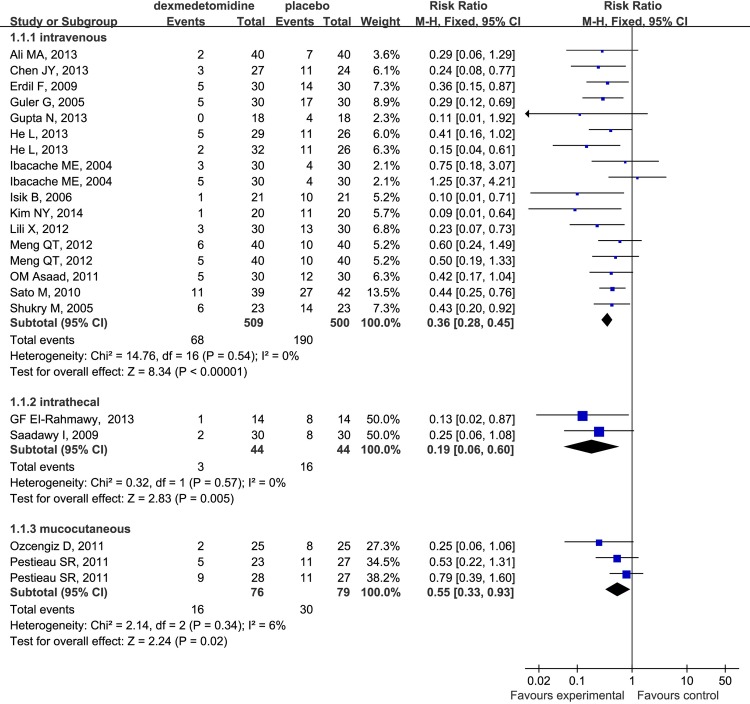
Forest plot for the effect of different administration ways of dexmedetomidine on the incidence of EA.

**Fig 5:**
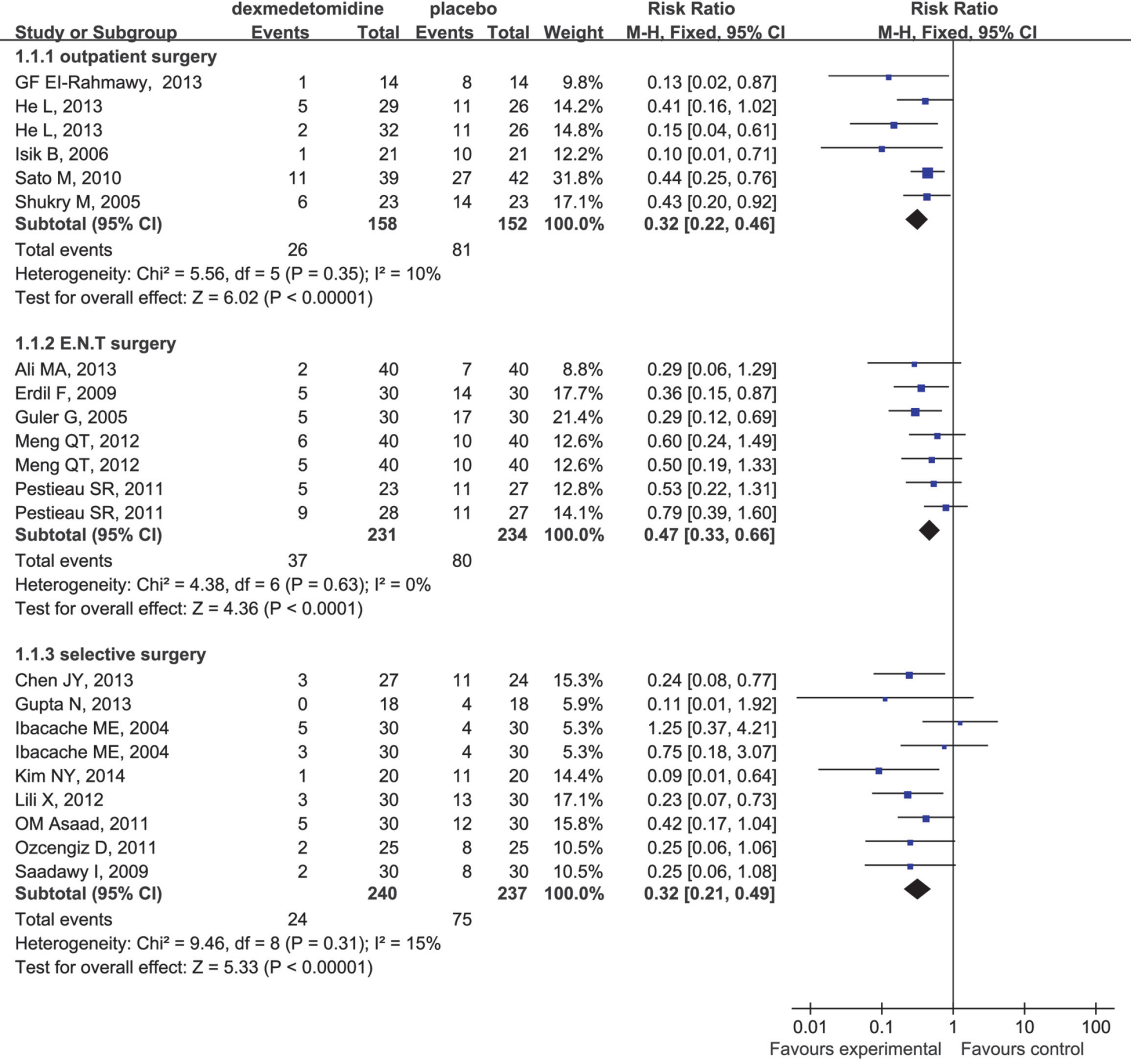
Forest plot for the effect of different surgery procedures on the incidence of EA.

**Fig 6:**
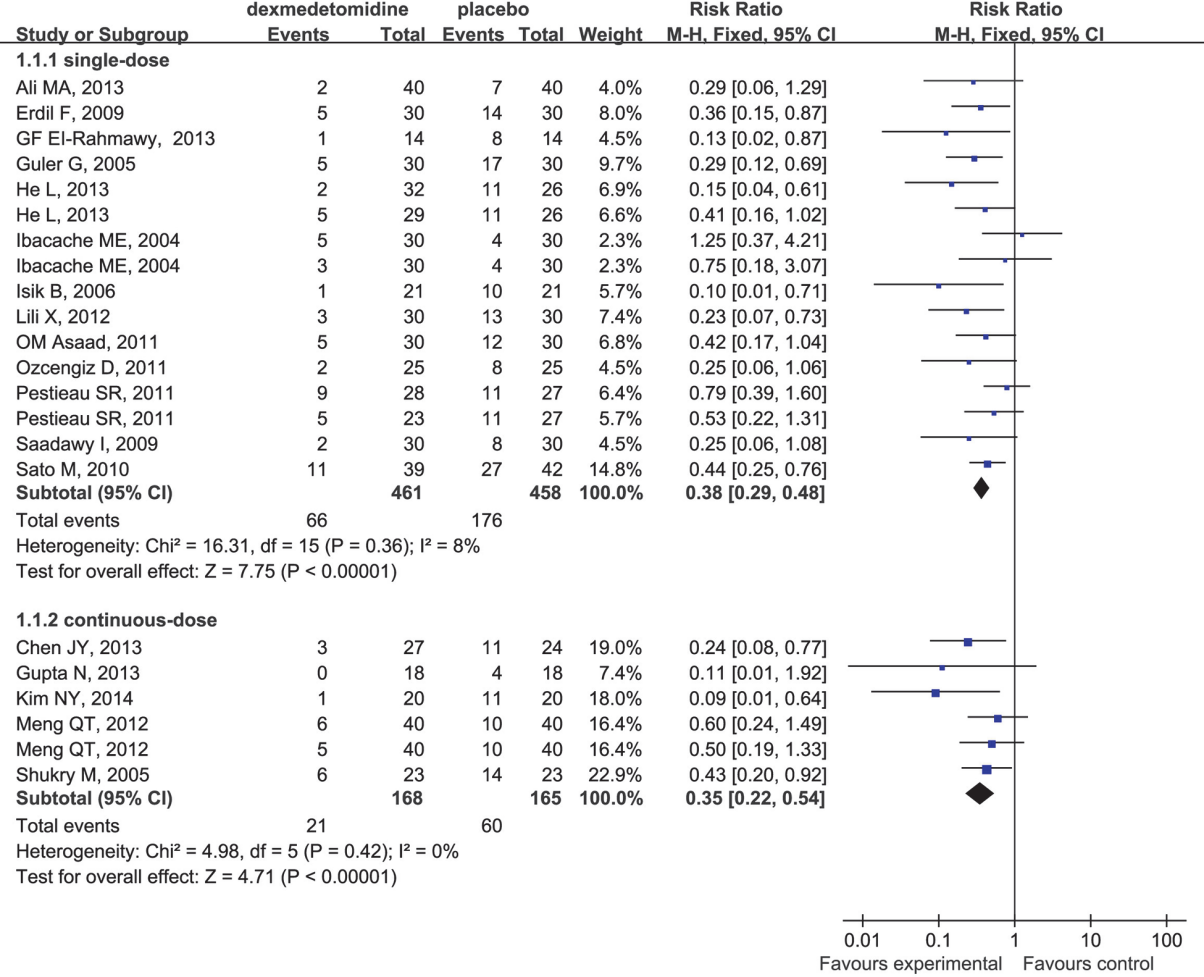
Forest plot for the effects of bolus or continuous-dosage administration of dexmedetomidine on the incidence of EA.

In the subgroup analysis, our study suggested that dexmedetomidine effectively decreased the incidence of EA regardless of the route when administered intravenously (RR 0.36; 95% CI 0.28 to 0.45), intrathecally (RR 0.19; 95% CI 0.06 to 0.60) and mucocutaneously (RR 0.55; 95% CI 0.33 to 0.93) (Fig 4). The same phenomenon was found to be associated with different surgical procedures (Fig 5) (outpatient surgery: RR 0.32; 95% CI 0.22 to 0.46; ear-nose-throat (E.N.T) surgery: RR 0.47; 95% CI 0.33 to 0.66; selective surgery: RR 0.32; 95% CI 0.21 to 0.49) and drug dosages (Fig 6) (single-dose: RR 0.38; 95% CI 0.29 to 0.48; continuouse-dose: RR 0.35; 95% CI 0.22 to 0.54).

**Fig 7:**
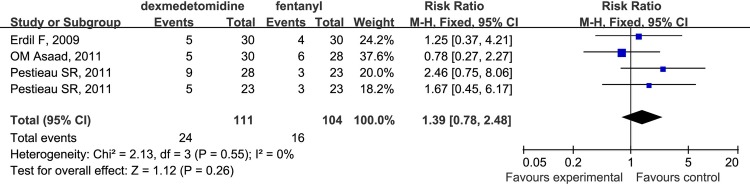
Forest plot for the incidence of EA. The plot displays the study, sample size, weighted risk ratio (RR), confidence interval (CI), and P value. Meta-analysis indicates dexmedetomidine had no difference on the incidence of EA compared with fentanyl. The square shown for each study (first author and year of publication) is the RR for individual trials, and the corresponding horizontal line is the 95% CI. The diamond is the pooled RR with the CI. The different sizes of squares indicate the weight the individual trials had in the analysis, taking into account sample size.

Moreover, there was no significant difference in the incidence of EA among patients anesthetized with dexmedetomidine or fentanyl (RR 1.39; 95% CI 0.78 to 2.48; Fig 7). It should be noted that this conclusion was based on the analysis of only three studies and therefore, should be interpreted with caution.

**Fig 8:**
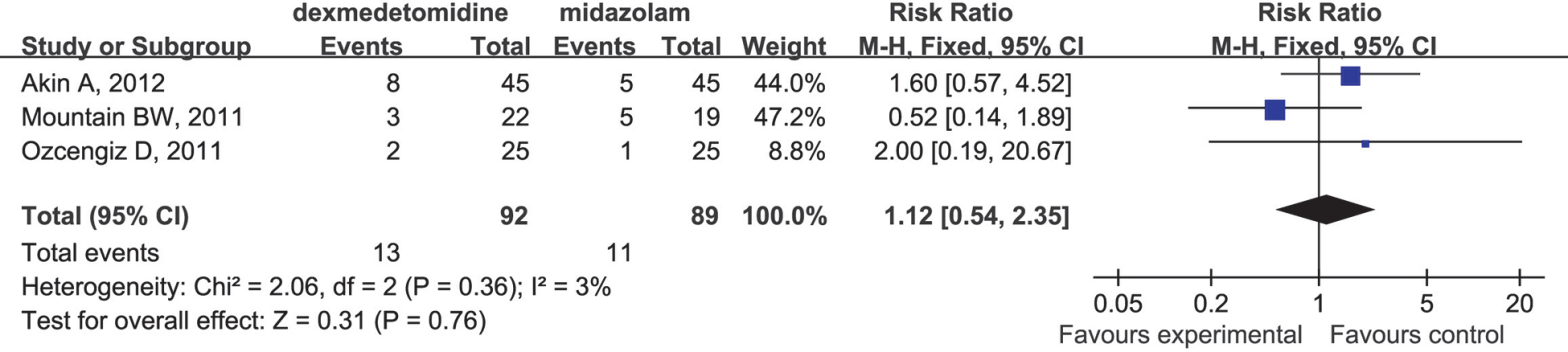
Forest plot for the incidence of EA. The plot displays the study, sample size, weighted risk ratio (RR), confidence interval (CI), and P value. Meta-analysis indicates dexmedetomidine had no difference on the incidence of EA compared with medizolam. The square shown for each study (first author and year of publication) is the RR for individual trials, and the corresponding horizontal line is the 95% CI. The diamond is the pooled RR with the CI. The different sizes of squares indicate the weight the individual trials had in the analysis, taking into account sample size.

Meanwhile, compared with midazolam (RR 1.12; 95% CI 0.54 to 2.35; Fig 8), dexmedetomidine had no significant impact on the incidence of EA, although this observation may also be limited by the small number of studies (only three) included in our analysis.

### Emergence Time

**Fig 9:**
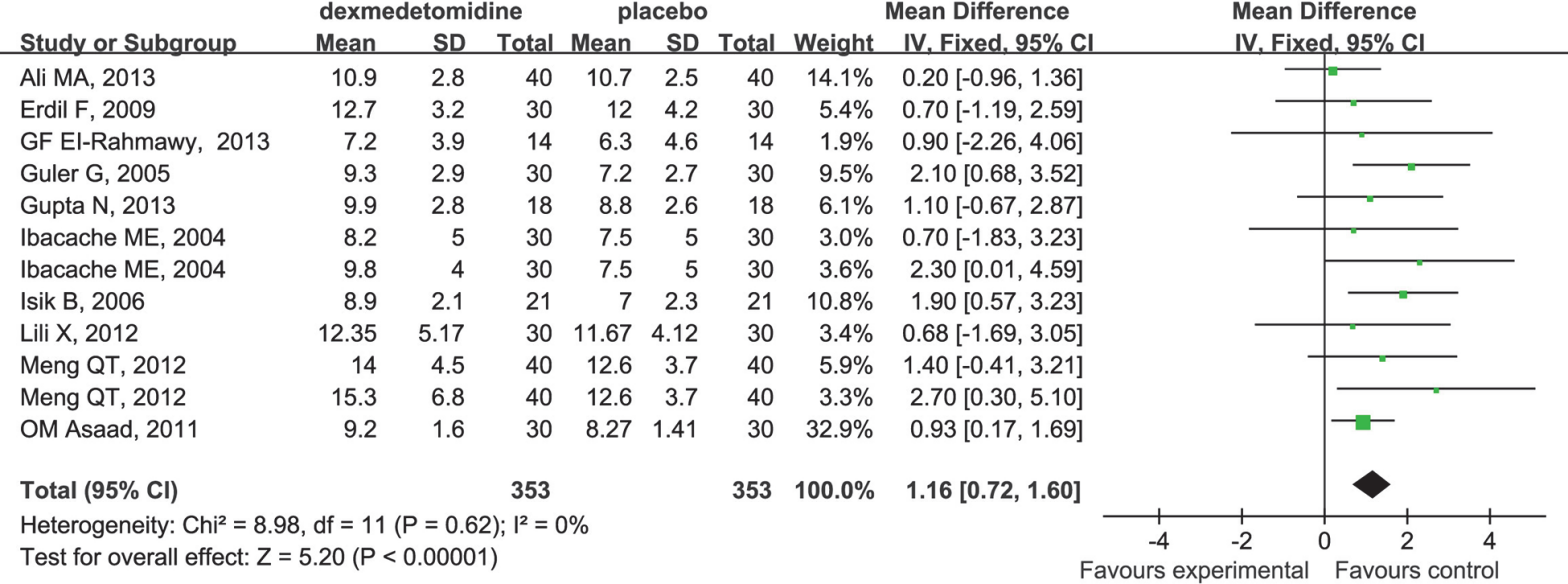
Forest plot for emergence time. The plot displays the study, sample size, weighted mean differences (WMD), confidence interval (CI), and P value. Meta-analysis indicates dexmedetomidine significantly increased emergence time compared with the placebo group. The square shown for each study (first author and year of publication) is the mean difference for individual trials, and the corresponding horizontal line is the 95% CI. The diamond is the pooled WMD with the CI. The different sizes of squares indicate the weight the individual trials had in the analysis, talking into account sample size and standard deviations.

Dexmedetomidine compared to placebo had a delayed effect on emergence time in children undergoing sevoflurane anesthesia (WMD 1.16; 95% CI 0.72 to 1.60; Fig 9).

### Time to Extubation

**Fig 10:**
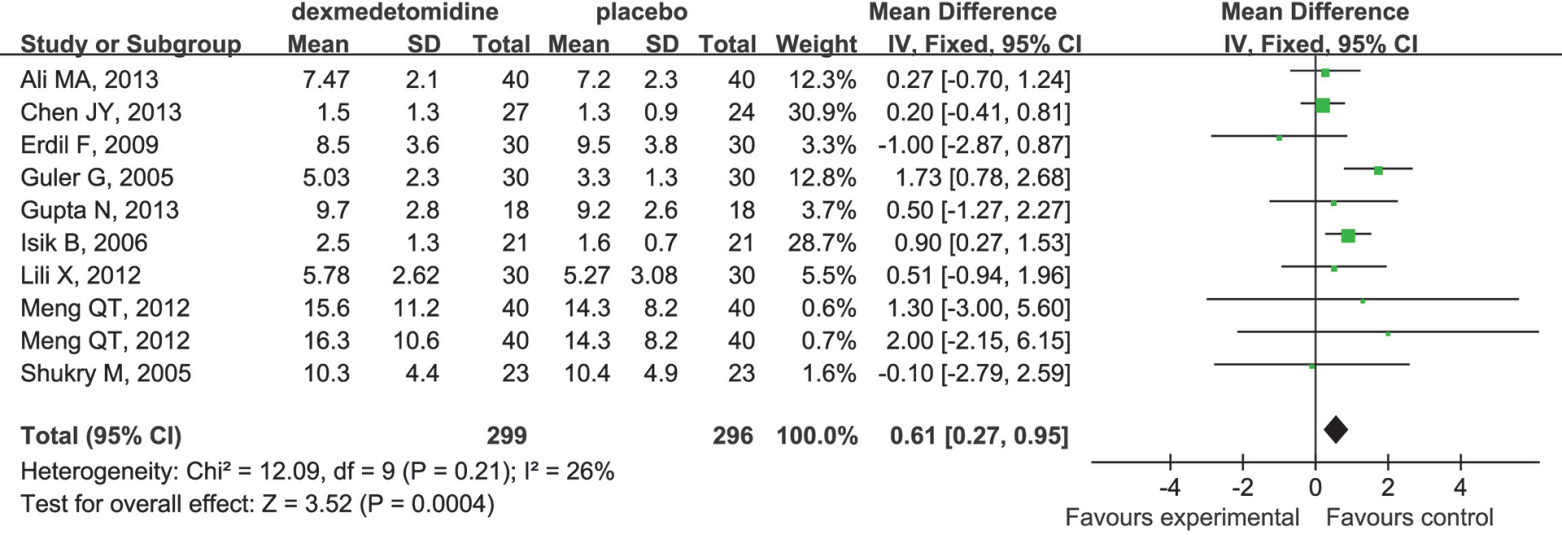
Forest plot for time to extubation. The plot displays the study, sample size, weighted mean differences (WMD), confidence interval, and P value. Meta-analysis indicates dexmedetomidine significant increased the time to extubation compared with the placebo group. The square shown for each study (first author and year of publication) is the mean difference for individual trials, and the corresponding horizontal line is the 95% CI. The diamond is the pooled WMD with the CI. The different sizes of squares indicate the weight the individual trials had in the analysis, talking into account sample size and standard deviations.

Dexmedetomidine compared to placebo increased the time to extubation in children undergoing sevoflurane anesthesia (WMD 0.61; 95% CI 0.27 to 0.95; Fig 10).

### Post-anesthesia Nausea and Vomiting

**Fig 11:**
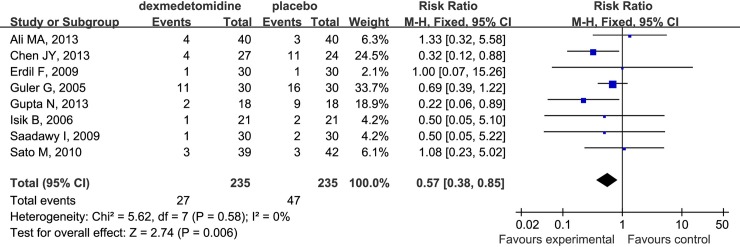
Forest plot for the number of patients with post-operative nausea and vomiting. The plot displays the study, sample size, weighted risk ratio (RR), confidence interval (CI), and P value. Meta-analysis indicates dexmedetomidine significantly decrease the number of patients with post-operation nausea and vomiting compared with the placebo group. The square shown for each study (first author and year of publication) is the RR for individual trials, and the corresponding horizontal line is the 95% CI. The diamond is the pooled RR with the CI. The different sizes of squares indicate the weight the individual trials had in the analysis, taking into account sample size.

Compared to placebo, dexmedetomidine decreased significantly the incidence of the occurrence of post-operative nausea and vomiting in children undergoing sevoflurane anesthesia (RR 0.57; 95% CI 0.38 to 0.85; Fig 11).

### Number of Patients Requiring Analgesic

**Fig 12:**
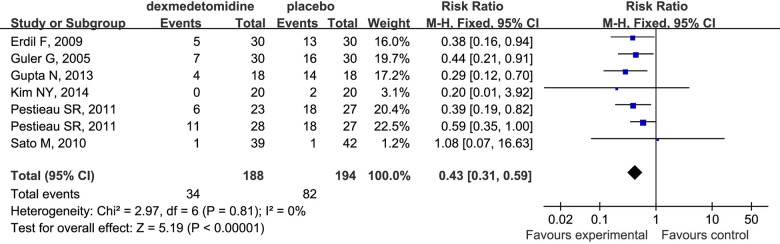
Forest plot for number of patients requiring analgesic. The plot displays the study, sample size, weighted risk ratio (RR), confidence interval (CI), and P value. Meta-analysis indicates dexmedetomidine significantly decreased the number of patients requiring analgesa compared with the placebo group. The square shown for each study (first author and year of publication) is the RR for individual trials, and the corresponding horizontal line is the 95% CI. The diamond is the pooled RR with the CI. The different sizes of squares indicate the weight the individual trials had in the analysis, taking into account sample size.

**Fig 13:**
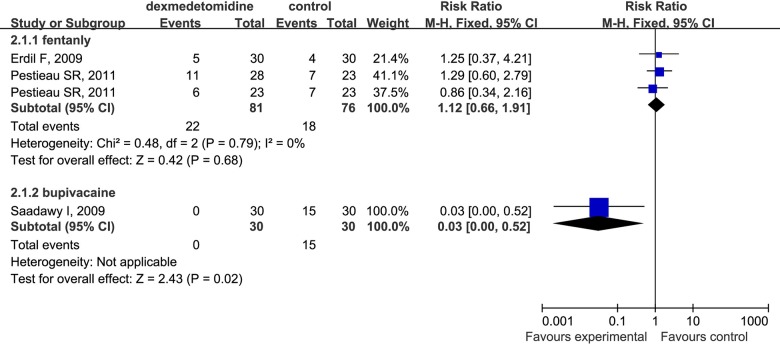
Forest plot for number of patients requiring analgesic dexmedetomidine vs Fentanyl and Bupivacaine. The plot displays the study, sample size, weighted risk ratio (RR), confidence interval (CI), and P value. Meta-analysis indicates the analgesia effect of dexmedetomidine on postoperation pain has no significantly statistical differences compared with fentanyl. The square shown for each study (first author and year of publication) is the RR for individual trials, and the corresponding horizontal line is the 95% CI. The diamond is the pooled RR with the CI. The different sizes of squares indicate the weight the individual trials had in the analysis, taking into account sample size.

Dexmedetomidine reduced the number of patients requiring analgesic compared to placebo in children undergoing sevoflurane anesthesia (RR 0.43; 95% CI 0.31 to 0.59 Fig 12). More importantly, dexmedetomidine had identical analgesic effects compared with fentanyl (RR 1.12; 95% CI 0.66 to 1.91 Fig 13) for the children under sevoflurane anesthesia. Furthermore, the required dosage of local anesthetics such as bupivacaine was reduced and the analgesia time was prolonged (Fig 13).

### Time to Discharge from Recovery Room

**Fig 14:**
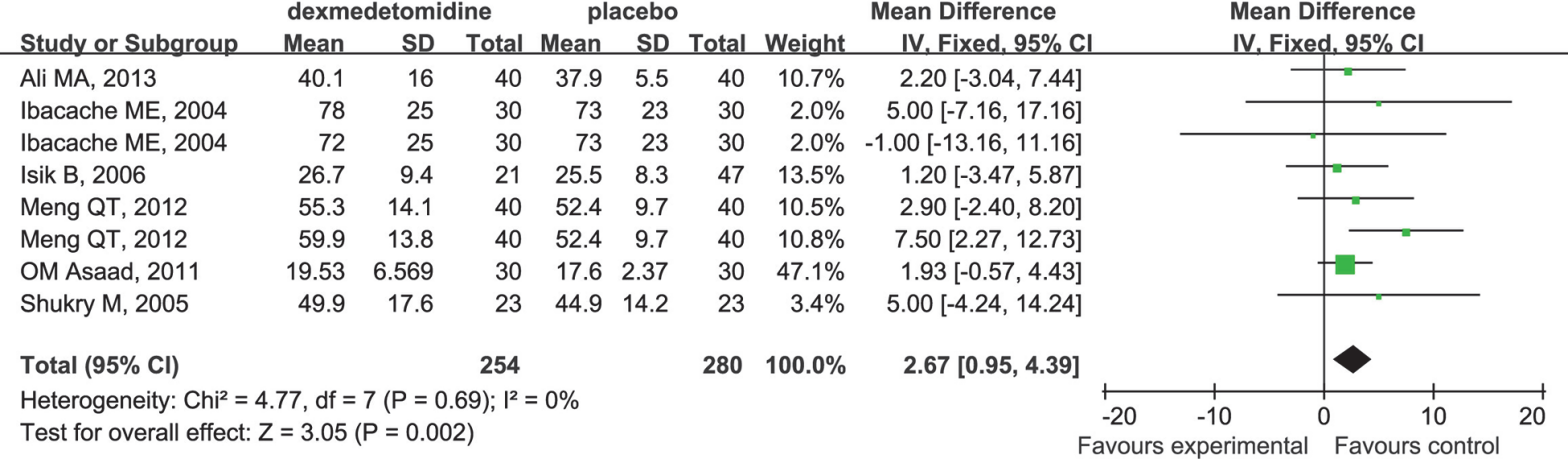
Forest plot for time to discharge from recovery room. The plot displays the study, sample size, weighted mean differences (WMD), confidence interval (CI), and P value. Meta-analysis indicates dexmedetomidine significant increase time to discharge from PACU compared with the placebo group. The square shown for each study (first author and year of publication) is the mean difference for individual trials, and the corresponding horizontal line is the 95% CI. The diamond is the pooled WMD with the CI. The different sizes of squares indicate the weight the individual trials had in the analysis, taking into account sample size and standard deviations

Dexmedetomidine compared to placebo increased the time to discharge from the recovery room in children undergoing sevoflurane anesthesia (WMD 5.61; 95% CI 4.28 to 6.94). Unfortunately, severe heterogeneity (I^2^ = 76%) was obtained in this analysis, which was mainly attributed to the study by Chen JY et al [13]. When this trial was excluded, the heterogeneity changed from 76% to 0%, and there was also a significant difference in the time to discharge from the recovery room in favor of dexmedetomidine (WMD 2.67; 95% CI 0.95 to 4.39; Fig 14).

## Discussion

Twenty RCTs [2,13–31] were identified to evaluate the effect of EA and recovery profiles associated with dexmedetomidine compared with placebo, fentanyl and midazolam after sevoflurane anesthesia in children. Nevertheless, there are no widely accepted assessment scales for the evaluation of EA in children, which led to the use of numberous assessment methods and agitation rating systems [13–34]. In most trials, the Pediatric Anesthesia Emergence Delirium scale (PAED) [35] was used for assessing the incidence of EA. We chose the time point after arrival to the PACU for assessment in this meta-analysis. In addition, we chose the incidence of EA post-sevoflurane anesthesia in children as a primary outcome measures analyzed in the present study. This analysis showed that compared with placebo, dexmedetomidine significantly decreased the incidence of EA in children undergoing sevoflurane anesthesia (RR 0.37; 95% CI 0.30 to 0.46). Zhang et al [36] suggested the effect of intravenous dexmedetomidine in the prevention of EA (RR 0.346; 95% CI 0.263 to 0.453). They also assessed the incidence of PONV from the entrance of PACU to 24 hours after surgery (WMD 4.597; 95%CI -0.080 to 9.275). However in their paper only 7 trials were examined, and they mentioned “when the trials of Gupta et al or Chen et al was removed, the reliability of the test would decreased”. In our paper, we assessed the incidence of PONV with more trials included (8 trials). We found that dexmedetomidine significantly decreased the incidence of occurrence of PONV in children undergoing sevoflurane anesthesia (RR 0.57; 95% CI 0.38 to 0.85). Furthermore, we did subgroup analysis for the different route of dexmedetomidine administration. We found that intrathecal and mucocutaneous administration also both decreased the incidence of EA (intrathecally: RR 0.19; 95% CI 0.06 to 0.60; mucocutaneously: RR 0.55; 95% CI 0.33 to 0.93), which would provide new and appropriate choice for dexmedetomidine administration in clinical work. This is also the difference between ours and the study of Sun et al [37]. In their study, they calculated the sevoflurane related EA in adenotonsillectomy (RR 0.351; 95% CI 0.033 to 0.422). Moreover, Dahmani et al [38] suggested that the E.N.T surgery is associated with a high incidence of EA. In our paper, we did performed the subgroup analysis on the different surgery procedures including outpatient surgeries, E.N.T. surgeries and selective surgeries, which provide more theoretical foundation for application of dexmedetomidine in clinical different surgeries. We found that dexmedetomidine also effectively prevented the EA associated with outpatient surgery (RR 0.32; 95% CI 0.22 to 0.46), E.N.T surgery (RR 0.47; 95% CI 0.33 to 0.66) and the selective operation (RR 0.32; 95% CI 0.21 to 0.49). This provided a novel strategy and broader application for preventing the occurrence and development of EA in children after sevoflurane anesthesia.

EA is a negative behavior including combative movements, excitability, thrashing, disorientation and inconsolable crying.[2,3]. It not only provides a challenging situation to post-anesthesia care providers but also results in higher rate of postoperative complications such as bleeding from operative sites and disturbing the recovery environment[39]. Early epidemiologic studies reported a 5.3% incidence of EA in postoperative patients [39], with a widely varying incidence of EA in children (10% to 80%) [40]. Although the precise etiology of EA following anesthesia is unknown, the risk factors including preschool age, pain, the use of inhalation anesthesia and otolaryngology procedures may provoke this phenomenon[4,40–42]. Meanwhile, studies found that a high incidence of EA was closely associated with sevoflurane anesthesia, and there has not been well-established prophylaxis and treatment [43–45]. Dexmedetomidine might be useful for management of post-anesthesia EA [18,21,22]. It is worth noting that the evidence of the effects of dexmedetomidine on EA and recovery profiles is still unclear.

Sevoflurane, an inhalation anesthesia agent, is commonly used in clinic anesthesia with rapid induction and fast recovery due to its low blood/gas partition coefficient (0.68) [46]. For the benefit of weak airway irritation, greater hemodynamic stability, comfortable quality and better cooperation with anesthetists, sevoflurane is popular choice for anesthesia in children [47]. However, some studies have found that a high incidence of EA (up to 80%) was associated with sevoflurane in children [44,48]. It is frustrating that the morbidity of sevoflurane-associated EA is not related to either the duration of exposure or dose [43]. Although a high incidence of sevoflurane-associated EA has been assumed to be largely related to rapid awakening, propofol anesthesia revealed rapid emergence properties were associated with a low incidence of EA [48]. Similarly, desflurane exhibited faster recovery than other inhalation agents, and has been found to be associated with a high incidence of EA in pediatric patients, which does not support the advantage of rapid emergence[49]. At present, the underlying mechanism of sevoflurane anesthesia-associated EA remains unclear. Further studies are necessary to determine the most effective way to manage EA in order to improve peri-operative outcome in pediatric patients.

Due to its pharmacologically unique profile, dexmedetomidine can produce stage 2 non-rapid eye movement sleep through activation of the endogenous sleep-promoting pathway, thereby inducing conscious sedation so that patients are drowsy but cooperative and aroused[50,51]. Meanwhile, dexmedetomidine can also produce its analgesic effect via receptors in the spinal cord and attenuate the stress response without evident respiratory depression [52]. In addition, because of the short half-life, about 1.5 to 3 hours, and lack of respiratory side effects, dexmedetomidine is usually superior to other hypnotics and barbiturates [53]. Therefore, based upon these characteristics dexmedetomidine, the pharmacologically active dextro-isomer of medetomidine [4,54], is extensively applied in clinic, especially for sedation and analgesia in pediatric patients [55,56]. Our analysis suggests that compared with midazolam, dexmedetomidine has no significant effect on the incidence of EA, although this conclusion is limited by the small number of studies (n = 3) (RR 1.12; 95% CI 0.54 to 2.35) and further research is required for confirmation. However, in terms of sedation score or anxiety score, dexmedetomidine had identical effects compared to midazolam[31]. It should be noted that dexmedetomidine decreased the side-effects induced by midazolam, such as delayed recovery time including the time to eye opening, verbal response and cooperation [57]. However, midazolam produced inferior postoperative analgesia compared with dexmedetomidine[31], indicating that dexmedetomidine is a better choice of sedation for pediatric patients.

Analgesia, one of main pharmacological properties of dexmedetomidine, is deemed to play an important role in preventing EA after inhalation anesthesia. Some studies found that although the pain alone could not cause EA [58,59], inadequate postoperative pain control may be closely associated with varying differences in the incidence of EA[60,61], therefore it is one of major causes of the increased frequency and severity of EA[6,62]. For children, it is too difficult to describe pain objectively, therefore various pain assessment tools such as the visual analogue scale (VAS) [63] and the objective pain scale (OPS) [64] were to compare the number of patients who needed post-anesthesia pain control. The results of this meta-analysis suggested that dexmedetomidine could significantly decrease the number of patients requiring post-anesthesia rescue analgesia (RR 0.43; 95% CI 0.31 to 0.59), which might be partly responsible for decreasing the incidence of EA. However, our analysis revealed that there was no significant difference in the incidence of EA induced by dexmedetomidine and fentanyl (RR 1.39; 95% CI 0.78 to 2.48). Although our analysis is subject to limitation by the small number of studies included (only three), the results may provide the basis of a new strategy for reducing postoperative pain in that compared with fentanyl, dexmedetomidine provided superior analgesia (RR 1.12; 95% CI 0.66 to 1.91). In addition to avoiding side-effects such as respiratory depression induced by opioid analgesics, dexmedetomidine significantly prolonged the duration of analgesia and decreased the total consumption of rescue analgesic compared with the use of local anesthetics alone [24]. Consequently, due to superior analgesia and fewer side-effects, dexmedetomidine is implicated as a substitute for opioid analgesics for pediatric patients.

Opioids are widely used for analgesia peri- and post-operatively. However, the adverse effects of opioids including emesis, excessive sedation and risk of respiratory depression restrict their clinical application [7]. Moreover the nausea and vomiting caused by opioids affects the recovery quality of children, and produces an intense environment for post-anesthesia care providers [3–5]. In our analysis, dexmedetomidine significantly decreased the number of patients with nausea and vomiting compared with placebo (RR 0.57; 95% CI 0.38 to 0.85). Therefore, the low incidence of nausea and vomiting might provide an advantage, by decreasing the risk of airway obstruction and increasing post-operation safety.

Some studies indicate that rapid emergence from anesthesia, especially inhalation anesthesia, might be a possible cause of EA [6]. In our analysis, dexmedetomidine significantly increased emergence time (WMD 1.16; 95% CI 0.72 to 1.60) and extubation time (WMD 0.61; 95% CI 0.27 to 0.95) in children undergoing sevoflurane anesthesia in PACU. This might be due to the excessive sedation associated with dexmedetomidine, and contribute to the decreased incidence of EA post-anesthesia. However, time to discharge from PACU in the dexmedetomidine group was also significantly increased compared with the placebo group in our study (WMD 2.67; 95% CI 0.95 to 4.39). The new measurement which could reverse such adverse effects of dexmedetomidine should be for the focus of future research.

The shortcomings of this meta-analysis are as follows: 1) some outcome measures data, such as agitation scores and pain scores, which were not normally distributed and were reported in the form of median and quartile, and therefore could not be included in the meta-analysis; 2) only English language reports have been included and consequently we may have missing data from important studies published in other languages.

## Conclusion

From this meta-analysis it is reasonable to conclude that according to the currently available data, intra-operative administration of dexmedetomidine statistically decreased the incidence of EA and postoperative pain in children undergoing sevoflurane anesthesia. Unlike opioids, which are associated with respiratory depression, dexmedetomidine could prove to have safer sedative and analgesic effects. Meanwhile, dexmedetomidine decreased the number of patients with vomiting, which might be an advantage for airway safety. However, compared with placebo, there was a significant increase in emergence time, time to extubation and discharge from recovery room. Further research is necessary to examine the proper dose, timing and period of dexmedetomidine infusion which could overcome the high incidence of EA induced by sevoflurane anesthesia in pediatrics with shorter emergence.

## Supporting Information

S1 PRISMA Checklist PRISMA Checklist. (DOC)
